# Visual Information Processing in Young and Older Adults

**DOI:** 10.3389/fnagi.2019.00116

**Published:** 2019-05-16

**Authors:** Deena Ebaid, Sheila G. Crewther

**Affiliations:** Department of Psychology and Counselling, School of Psychology and Public Health, La Trobe University, Melbourne Campus, VIC, Australia

**Keywords:** visual information processing, visual attention, attentional networks, processing speed, aging, cognitive processing

## Abstract

Decline in information processing with age is well-documented in the scientific literature. However, some discrepancy remains in relation to which cognitive domains are most susceptible to the aging process and which may remain intact. Furthermore, information processing has not been investigated nor considered as a function of affect, familiarity and complexity of tasks in a single experimental study. Thus, the current study investigated rate of visual information processing in 67 young university students (*M* age = 19.64 years) and 33 educated healthy older adults (*M* age = 70.33 years), while accounting for depression, anxiety and stress symptoms using the DASS. Rates of visual processing were measured as minimum time of stimulus exposure duration required for correct object recognition on a simple visual task [Inspection Time (IT)], and on a more complex visual cognitive task known as Change Detection (CD)] as well as words per minute on a text reading task (FastaReada). The results demonstrated significantly slower performance by older adults on the IT and CD, but comparable rates of text reading on a semantically more complex, but ecologically valid and familiar visual task that requires organized sequential shifts in attention *via* eye movements, continuous visual processing, access to working memory and semantic comprehension. The results also demonstrated that affective influences did not play a role in the older adults task performance, and that changes in cognitive domains may begin with older adults being slower to attend to and identify newly appearing familiar objects, as well as slower to encode and embed new information in memory during tasks that require a less practiced/familiar task strategy.

## Introduction

A decline in cognitive functions including attention, speed of visual information processing, working memory, and dual task performance associated with the normal aging process is commonly reported in the scientific literature (Salthouse, 1996; Bashore et al., 1997; Hedden and Gabrieli, 2004; Finkel et al., 2007; Deary et al., 2009; Eckert, 2011; Lu et al., 2011; Cona et al., 2013; Harada et al., 2013; Ritchie et al., 2014; Ebaid et al., 2017; Ebaid and Crewther, 2018). Indeed, affective factors such as elevated levels of depression and anxiety symptoms are reported to exacerbate such decline (Beaudreau and O’Hara, 2008, 2009; Hammar and Ardal, 2009) while higher levels of education are often reported to mitigate cognitive decline in healthy older adults (Elgamal et al., 2011; Archer et al., 2018). Some cognitive abilities reliant on acquired knowledge (primarily verbal or language based) and defined by Cattell (1941) as Crystallized Intelligence (Gc), are reported to remain intact or even improve with increased age (Baltes et al., 1995; Hedden et al., 2005; Elgamal et al., 2011). By comparison, Fluid Intelligence (Gf) that includes skills such as inductive and deductive reasoning, problem solving, and manipulation of new information, are reportedly most sensitive to age-related declines (Cattell, 1963; Horn and Cattell, 1966).

Much of the impairments in cognitive function measured with sensory (visual and auditory) tasks are exacerbated by morphological and physiological changes in auditory function (Jayakody et al., 2018) and the eye [i.e., retinal integrity (reviewed in Brown et al., 2018)]. Brown et al. (2018) also demonstrated that conduction rates of visual information to cortex, and activation of visual attention as measured by decreases in flicker fusion thresholds, decline with age. A review by Wayne and Johnsrude (2015) also attests to the correlation between hearing loss and neurocognitive function and seems to support the decline in cognitive function in healthy aging that Salthouse (1996) has associated with a slower processing speed. Indeed, Salthouse’ *Processing Speed Theory of Adult Age Differences in Cognition*, has become influential across the aging literature in providing an underlying basis for the decline observed in other complex cognitive abilities (i.e., Bashore et al., 1997; Zimprich and Martin, 2002; Salthouse and Ferrer-Caja, 2003; Lemke and Zimprich, 2005; Finkel et al., 2007; Eckert, 2011; Lu et al., 2011; Ebaid et al., 2017).

Impaired sensory processing i.e., visual and auditory processing has also gained substantial attention in providing explanation for deficits in cognitive processing across the lifespan (Baltes and Lindenberger, 1997; Schneider and Pichora-Fuller, 2000; Bowl and Dawson, 2015; Peelle and Wingfield, 2016; Uchida et al., 2019), and this argument has been incorporated into several other theories of cognitive aging such as the *Sensory Deprivation Hypothesis, the Common-Cause Hypothesis* (Lindenberger and Baltes, 1994; Baltes and Lindenberger, 1997), and *the Information Degradation Hypothesis* (Schneider and Pichora-Fuller, 2000). Such theories collectively suggest a strong interaction between declines in sensory ability i.e., impaired vision and audition with age, and declines in cognitive abilities (for a review, see Roberts and Allen, 2016). Indeed, the first study to explicitly control for such age-related sensory decline and measure cognitive performance on an auditory digit span task was conducted by Füllgrabe et al. (2015), who used a sample of audiometrically matched healthy young and older participants. Participants were also matched on age-corrected performance IQ scores and years of education, and did not demonstrate any significant differences in auditory digit span performance between older and younger adults (Füllgrabe et al., 2015).

Other psychological explanations of cognitive decline with aging such as the *Inhibitory Deficit Hypothesis* (Hasher and Zacks, 1988) suggest that older adults may perform worse on cognitive tasks compared to younger populations because they are more susceptible to irrelevant stimuli and have greater difficulty inhibiting distractions, resulting in heightened distractibility, poorer retrieval of task-relevant details, and overall worsened task performance. A further hypothesis derived from a *disuse perspective* (Salthouse, 1991), posits that differences in cognitive performance between age groups are at least in part due to changes in the nature of the activities performed or *not* performed by people of various age groups. Such ideas were conceptualized as early as the 1930s when Sorenson (1933, p. 736) suggested that *“A decrease in test ability probably is caused by the fact that adults, as they grow older, exercise their minds less and less with materials found on psychological tests.”*

Visual attention and information processing are particularly relevant to the study of cognitive decline as most human behavior is visually driven (Mundinano et al., 2018), with visual attention considered the key driver of most perceptual, cognitive, and behavioral process (Godefroy et al., 2010; Crewther et al., 2013). Visual attention has often been conceptualized as three cognitive networks that carry out the functions of alerting, orienting and executive control (Posner and Petersen, 1990; Petersen and Posner, 2012) where alerting is defined as achieving and maintaining an alert state, orienting as the selection of information for sensory input, and executive control is defined as resolving conflicting information among responses and about task-relevant stimuli (Posner and Petersen, 1990; Fan et al., 2002; Petersen and Posner, 2012). Whether attention is captured in a “bottom up” or a “top-down” manner has been suggested to affect cognitive task performance by older adults. Specifically, top-down or goal-directed control of attention is facilitated by an individual’s internal valuations, goals, or perceptual-sets and studies have reported that when tasks are reliant on top-down control of attention, older adults perform as fast and as accurately as older adults (Madden, 2007). On the other hand, when visual tasks require bottom-up control of attention, i.e., inhibition of distractors and are driven by salient differences among the features of the stimuli, older adults typically demonstrate a slower response rate or reaction time (Madden, 2007). Such findings have led to the conclusion that bottom-up control of attention is most susceptible to age-related decrements while top-down control of visual attention remains relatively intact across the lifespan (Madden, 2007).

Behavioral data using the Attention Network Task (ANT; Fan et al., 2002) has demonstrated that healthy older adults have less efficient attentional networks compared to younger adults (Jennings et al., 2007; Gamboz et al., 2010; Mahoney et al., 2010; Kaufman et al., 2016), however discrepency in findings still remain (MacLeod et al., 2010). For example, this was recently assessed in a study conducted by Kaufman et al. (2016) who reported slower button-press reaction times for older adults across all three attention networks, though after controlling for generalized slowing, only the alerting system remained significantly reduced. However, button-press reaction times are problematic when measuring domains of cognitive speed in a healthy aging population, given the potential contamination of motor slowing that may be contributing to seemingly slower task performance (Ritchie et al., 2014; Ebaid et al., 2017), and this is not often accounted for in the aging literature.

To date, visual information processing in healthy older adults in terms of rate of information processing in the context of Posners three attentional networks (Posner and Petersen, 1990; Petersen and Posner, 2012) has not been investigated especially while accounting for affective influences, complexity and familiarity of tasks in a single experimental study. Visual tasks varying in complexity are necessary to determine which aspect of attentional control and cognitive processing is most susceptible to the aging process and whether any may remain intact. More specifically, if older adults are slower at identifying visual stimuli without any excess demands on working memory, as in a simple object recognition task requiring conscious access to visual perception and minimal decision making demands, then it is likely that older adults will also be slower when faced with the more complex task of change detection (CD), that requires embedding in short-term memory of several objects in a visual array prior to decision making of same/different comparison of a second array presented soon after. Furthermore, in addition to such tasks, it is unknown whether a cognitively complex visual task that is more familiar and ecologically valid, i.e., reading, will elicit similar age-group differences.

Thus, the current study aimed to compare the rate of visual information processing (specifically, threshold exposure time) in young and older adults of similar educational backgrounds, using computerized perceptual speed tasks varying in complexity and familiarity, while considering depression, anxiety, and stress symptoms. We aimed to measure rate of visual information processing and attention using an Inspection Time (IT) task requiring object recognition, a CD task, and a rapid reading task known as the FastaReada which is an ecologically valid measure of visual attention and processing amongst an educated population (Hecht et al., 2004; Elhassan et al., 2015). Each of our three tasks requires alerting, orientation and executive control of attention, though to different degrees. Based on the results from Ebaid et al. (2017), it was hypothesized that young and older adults would again demonstrate comparable results on the simple visual perceptual IT task but that younger adults would perform faster than older adults on the more complex CD task, given the additional requirement for executive control of attention, new learning, and memory. We also hypothesized that older adults would demonstrate a slower rate of reading than the young adults on the cognitively complex FastaReada task due to excess demands on all attentional networks and for fast visual processing of stimuli prior to comprehension of text, even though fast and fluent reading is likely to be a familiar and well-practiced task amongst an educated sample.

## Materials and Methods

### Participants

The sample included 100 healthy participants who were derived from a larger pool of participants from previous studies conducted by our lab (Ebaid et al., 2017; Ebaid and Crewther, 2018) and were divided into a young adult and older adult group. The younger group included 67 individuals between the age of 18–29 years (*M* age = 19.64, SD = 2.26) and comprised 59 females, and eight males, while the older group included 33 individuals of whom 25 were females and eight were males between the age of 60–81 years (*M* age = 70.33, SD = 5.62). Younger adults were recruited from La Trobe University, Melbourne, and consisted of 1st year Psychology students who received course credit, while the older adults were recruited through the University of the Third Age (U3A) and received a $20 Coles-Myer voucher for participation. U3A is an international volunteer organization where interested older individuals attend for their own interest and not for any qualifications (for more information please visit^1^). Both groups had tertiary education with the younger sample reporting an average of 7.64 years (SD = 1.06), and the older sample had an average of 10.91 years (SD = 3.38) of post-primary education, with the number of years of formal education recorded from the 1st year of secondary school onwards. All participants had normal or corrected-to-normal visual acuity whereby 6/6 visual acuity was ensured. Participants self-reported whether volume on computerized tasks was adequate for comprehension, and for performance on the auditory digit span task. Participants who wore hearing aids were able to keep them in during the study. Pure-tone or speech audiometry was not explicitly assessed as all experimental tasks were visually based and were all presented at suprathreshold visual contrast. Exclusion criteria included the previous diagnosis of a neurological disorder and the inability to hear, understand and/or read in English with basic competence. A demographic questionnaire elicited information on age and education level. This study was carried out in accordance with the recommendations of the National Statement on Ethical Conduct in Human Research, La Trobe University Human Ethics Committee (UHEC) and all subjects gave written informed consent in accordance with the Declaration of Helsinki. The protocol was approved by La Trobe University Human Ethics Committee, approval number S15/19.

### Materials

#### Screening Measures

##### Depression Anxiety and Stress Scale DASS-21 (Lovibond and Lovibond, 1995)

The Depression Anxiety and Stress Scale (DASS-21; Lovibond and Lovibond, 1995) is a 21-item self-report instrument that measures negative emotional states relating to depression, anxiety and stress. The DASS-21 was administered as a screening tool to identify whether participants had negative emotional states beyond a “mild” level (i.e., depression >6, anxiety >5, or stress >9) that might independently affect performance on the cognitive tasks (Beaudreau and O’Hara, 2008, 2009).

##### Auditory and Visual Backward Digit Span (DSB) Task as a Correlative Measure of Intellectual Functioning

Automated auditory and visual backward digit span tasks were administered to assess working memory capacity as a correlative measure of intellectual functioning (Lichtenberger and Kaufman, 2009) on the basis that working memory capacity has previously been reported to positively correlate with Full Scale IQ (Griffin and Heffernan, 1983; Salthouse and Pink, 2008), fluid intelligence and executive functioning (Cowan et al., 2005; Fukuda et al., 2010). Scores on the DSB were compared against appropriate age and education level norms.

The DSB tasks adapted from the Auditory Digit Span subset of the Wechsler Adult Intelligence Scale 4th Edition (WAIS; Wechsler, 2008a,b) using Authorware Professional Software, and administered in both auditory and visual conditions on an Apple iMac (Retina 4K) computer with a 21.5-inch monitor. In the auditory condition, a series of random digits ranging from 1 to 9 were verbally presented to participants *via* a voice over on loudspeaker and participants were required to type the digits back in the *reverse* order using a keyboard. The auditory version has no visual representation of the numbers on the screen. In the *visual* digit span condition, digits were presented in black Ariel 92 pt font against a white background, at a rate of one digit per second, with no sound/voiceover reading the numbers. If successful, the participant was given a longer sequence. The number sequence began at a span length of two, and increased by one every two trials, i.e., participants were given two trials per span length. The task ceased when two consecutive responses were entered incorrectly, and the previously correct span length was recorded as the participants “backward digit span capacity.” Volume was set to the preferred level chosen by the participant after adjustment during the instructions phase, prior to formal commencement of the task.

### Experimental Measures

#### Inspection Time

**Figure 1:**
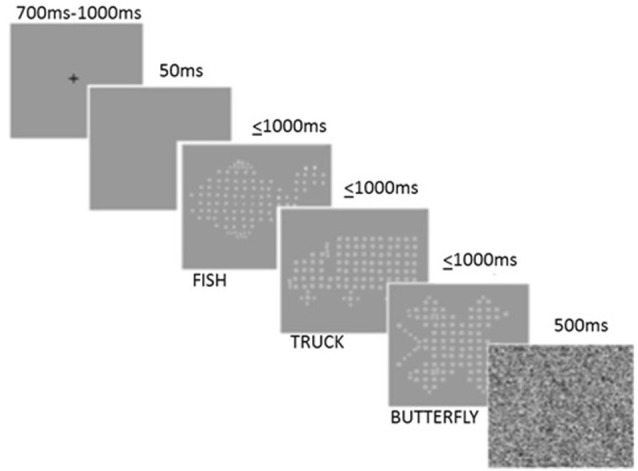
Modified Inspection Time (IT) task trial. Note: only one target stimuli of fish, truck, or butterfly is presented per trial.

A modified IT task, based on the version of Vickers et al. (1972), was adapted using Vpixx^2^ by Brown and Crewther (2012) and was used as a measure of early cortical perceptual speed by estimating threshold exposure duration required to successfully discriminate and identify a familiar visual stimulus, without any confound of motor reaction time thus ensuring that task performance is not confounded by age-related motor slowing (Ebaid et al., 2017). The task employs an inbuilt Visual Parameter Estimation by Sequential Testing (VPEST) algorithm, designed to estimate the exposure threshold required to discriminate and identify which of the three possible stimuli consisting of either a fish, truck or butterfly was presented (see Figure 1). The task was presented at suprathreshold contrast (well above each individuals contrast threshold) on an Apple eMac computer running at 89 Hz screen refresh rate. Participants were required to identify the target stimulus from the three options (fish, truck or butterfly) by manually responding on a keyboard after the stimulus had disappeared. Prior to each trial, a fixation cross was presented for a random duration between 700 and 1,000 ms, followed by a blank screen for 50 ms, after which the target stimulus was presented for variable exposure times for no greater than 1,000 ms. Presentation of the target stimulus was immediately followed by a mask, presented for 500 ms. The start of the next trial was triggered 100 ms after the termination of the mask. One thousand milliseconds was the maximum exposure time for any target stimulus. Confidence intervals and estimations of exposure time were calculated as part of the Vpixx program, where the lowest reoccurring exposure time is used to estimate and indicate the threshold duration of each participant’s perceptual time required to discriminate and accurately identify visual stimuli, with a shorter threshold exposure time indicative of a faster speed of processing of visual information; see Figure 1 for example of task.

### Change Detection

**Figure 2:**
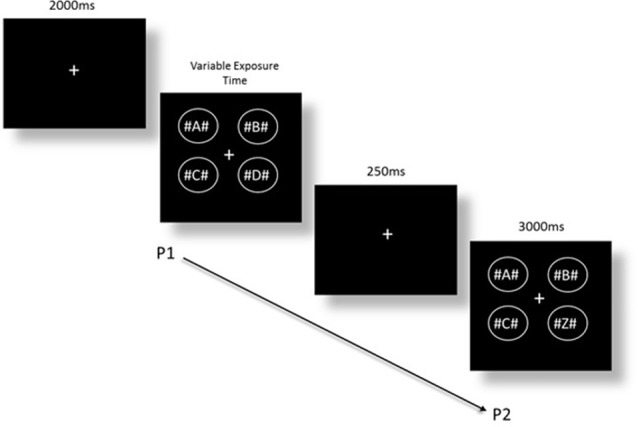
Change detection (CD) task where a change has occurred from presentation 1 (P1) to presentation 2 (P2).

The CD task was based on the Becker et al. (2000) version, as adapted by Rutkowski et al. (2003). The task consisted of the same software, computer, and VPEST technique as the modified IT task. The stimuli were four different alphabetic letters with a hash (#) symbol on either side of each letter, contained in a circle. The four circles were arranged into a square shape (see Figure 2). The addition of hash symbols around each letter was included to create visual crowding (Whitney and Levi, 2011), and as non-alphabetic task-irrelevant stimuli, as the four alphabetic letters alone in a single array is within the limit of visual-short term memory capacity (Cowan, 2001). A fixation cross was presented for 2 s at the start of each trial, followed by the first stimulus array of four letters for variable exposure times, and immediately followed by a 250 ms delay, and then another array of four letters (presented for a period of 3 s). In the *change* condition, one of the four letters was changed in the final presentation. In the *no-change* condition, the exact same letters were shown in both presentations. The two conditions were presented in random order. Participants were asked to indicate whether there was a *change* or *no-change* after each set of visual arrays, and the estimated exposure time for detection of change between two visual arrays was calculated. Again, the task does not measure motor reaction time nor is reliant on time taken to make a response, thus ensuring that task performance is not confounded by age-based motor speed (Ebaid et al., 2017). Confidence intervals and threshold estimations of exposure time were calculated by Vpixx where a lower estimated exposure time indicated a faster threshold response time required to detect change between visual stimuli. An example of the task is shown in Figure 2.

### FastaReada

FastaReada is a customized computer-generated task formulated using VPixx^3^ (Hecht et al., 2004; Elhassan et al., 2015) and designed to measure reading fluency by determining the number of words an individual can accurately read per minute. The task is reliant on visual attention, rapid processing of visual information, working memory, continuous inhibition of distractors, continuous access to lexical storage, integration of sublexical, orthographic, phonological, and lexico-semantic information to achieve fast semantic understanding of the text (Daneman and Hannon, 2001; Cotton and Crewther, 2009; Elhassan et al., 2015). During the task, an excerpt from a children’s Penguin novel (permission received from Penguin Group) was presented on a computer screen in Lucida Grande font, in 60 pt, six words at a time in narrative order. Presentation time was controlled *via* the PEST adaptive staircase algorithm based on a maximum-likelihood threshold estimation. Participants were asked to read the words out loud as accurately as possible and were informed that presentation will eventually become so short (i.e., in order to measure thresholds), that reading the six words on the screen would be impossible. Participants were encouraged to attempt this to the best of their ability. After each trial (i.e., after each set of six words), the investigator indicated accurate or inaccurate decoding by pressing a key on the keyboard, and this triggered the next presentation of words. Similar to the IT and CD tasks, the FastaReada does not measure response time nor reaction time. The score obtained at the end of the task provided an estimation of the number of words the participant can accurately read per minute.

### Procedure

Total testing time was approximately 1 h. The CD and IT tasks were preceded by five practice trials. To ensure accurate results for the CD and IT, the task was re-done if the confidence interval as indicated by the Vpixx data output was below 80%. The FastaReada task was not preceded with a practice trial as this was likely to confound performance on the task if the participant had knowledge about the upcoming text on the screen. All testing was conducted in a quiet room either at La Trobe University or U3A, where only the participant and experimenter were present.

### Data analysis

All analyses were performed using SPSS v. 25.0 (IBM Corp., Armonk, NY, USA). Data were screened for outliers on individual tasks and for any result indicating inconsistent performance across tasks. None were found.

## Results

### Relationships Between Negative Affect and Performance on the IT, CD, and FastaReada

The results revealed that DASS scores for each subcategory were less than “mild” and were in the normal range for both young and older adults. Interestingly, mean DASS scores for the older adults were lower than those in the younger population across all three subcomponents. Descriptive statistics are reported in Table 1. Correlation analyses also revealed no significant relationships between depression, anxiety or stress symptoms and performance on the IT, CD, or FastaReada in the older sample. In the younger sample, correlation analyses revealed a marginal significant positive correlation between depression scores and performance on the CD task, indicating that increased depression scores were correlated with a higher (i.e., slower) threshold estimation time to detect change between two visual arrays. The correlation matrix is presented in Table 2.

**Table 1:** Descriptive statistics of age, gender, negative affect (DASS), and backward digit span for young and older adults.

Age (Years)								
	Younger adults (*M* = 8, *F* = 59)				Older adults (*M* = 8, *F* = 25)			
	*N*	Range	*M*	SD	*N*	Range	*M*	SD
	67	18–29	19.64	2.26	33	60–81	70.33	5.62
**Years of Formal Education**								
	67	7–11	7.64	1.06	33	7–21	10.91	3.38
**Scores on Depression Anxiety Stress Scale (DASS-21)**								
DASS.dep	67	0–11	3.09	2.51	33	0–8	1.88	2.42
DASS.anx	67	0–12	3.34	3.03	33	0–7	1.79	1.93
DASS.str	67	0–14	5.89	3.49	33	0–13	3.91	2.88
**Scores on Backward Digit-Span (DSB) task**								
DSB.Aud	67	3–8	5.88	1.87	33	3–7	5.06	1.12
DSB.Vis	67	3–8	5.72	1.23	33	2–7	5.27	1.28

*Note: DASS.dep, DASS-21 depression score; DASS.anx, DASS-21 anxiety score; DASS.str, DASS-21 stress score; DSB.Aud; Auditory Digit Span Backward condition*.

**Table 2:** Correlation coefficients (*r*) for depression, anxiety, stress scores and performance on IT, CD, and FastaReada for young and older adults.

Younger Adults						
Measure	DASS.dep	DASS.anx	DASS-str	IT	CD	FastaReada
DASS.dep	-					
DASS.anx	0.522**	-				
DASS.str	0.541**	0.569**	-			
IT	−0.097	−0.070	−0.112	−	
CD	0.281*	0.097	0.031	−0.116	−	
FastaReada	−0.139	0.067	−0.002	−0.209	−0.191	−
**Older Adults**						
DASS.dep	−					
DASS.anx	0.599**	−				
DASS.str	0.546**	0.616**	−		
IT	0.007	0.025	−0.090	−		
CD	0.233	−0.062	0.106	0.054	−	
FastaReada	−0.117	0.083	0.216	0.089	−0.279	−

Note: DASS.dep, DASS-21 depression score; DASS.anx, DASS-21 anxiety score; DASS.str, DASS-21 stress score; IT, Inspection Time; CD, Change Detection. **p = < 0.01, *p< 0.05.

### Backward Digit-Span (DSB) as a Correlative Measure of Intellectual Functioning

Table 1 provides descriptive data of DSB scores for young and older adults. Normative data as indicated by the WAIS manual (Wechsler, 2008a,b) has been provided for healthy older adults between the ages of 20–24 years, 25–29 years, 65–69, and 70–74 and are mean DSB scores of 5.1, 4.9, 4.5, 4.3, and 4.4 respectively. Similar normative scores were noted in a recent study conducted by Monaco et al. (2013) aimed at providing standardization and normative data for the digit span tasks in a large healthy sample, which suggested the following norms for the DSB for healthy adults between 20 and 30; *M* = 5.07, between 61 and 70, *M* = 4.15, between 71 and 80 = 3.92. Thus, as shown in Table 1, young and older adults from the current study obtained scores that exceeded these suggested normative scores.

### Age-Group Differences in Performance on the IT, CD, and FastaReada

Significant differences in performance between young and older adults were demonstrated on the IT, where younger adults performed significantly faster than older adults (*M* difference = −87.335 ms, *t*_(74)_ = −7.214, *p* = < 0.001, *η*^2^ = 0.413) as well as on the CD, where task performance was significantly faster for younger adults than older adults (*M* difference* =* −258.488 ms, *t*_(77)_ = −3.348 *p* = 0.001, *η*^2^ = 0.127). There were no significant differences in FastaReada scores between young and older adults *t*_(97)_ = 0.336, *p* = 0.738, *η*^2^ = 0.001. These results are presented in Table 3.

**Table 3:** Descriptive statistics and independent samples *T*-test for mean difference for scores on the IT, CD, and FastaReada in younger and older adults.

	Younger adults				Older adults				Age-Group differences	
Measure	*N*	Range	*M*	SD	*N*	Range	*M*	SD	*p*	*η*^2^
IT (ms)	46	20.00–130.00	49.30	169.06	30	34.90–343.00	136.64	74.93	<0.001	0.413
CD (ms)	47	110.00–1288.00	636.36	338.52	33	175.80–1798.00	894.84	363.20	0.001	0.127
FastaReada	67	105.30–848.80	375.82	161.73	33	128.50–721.50	364.53	145.92	0.738	0.001

*Note: IT, Inspection Time; CD, Change Detection*.

### Relationships Among Age and Performance on the IT, CD, and FastaReada

As our age distribution was not normally distributed and included two distinct age groups, correlational analyses using Spearman’s Rank-Order Correlation were performed to investigate the strength, direction and significance of associations between age and dependent measures. Results revealed significant weak-moderate positive correlations between increasing age and threshold performance on IT (*r*_s_ = 0.569, *p* < 0.001) as well as age and threshold performance on CD (*r*_s_ = 0.382, *p* < 0.001). No significant correlations were revealed between age and performance on the FastaReada task. A full correlation table of all dependent measures is shown in Table 4.

**Table 4:** Spearman’s rank-order correlations between age, and scores on it, cd, and FastaReada.

Measure	Age	IT	CD	FastaReada
Age	−			
IT	0.569**	−		
CD	0.382**	0.143	−	
FastaReada	−0.014	−0.063	−0.205	−

Note: IT, Inspection Time; CD, Change Detection, **p< 0.01.

## Discussion

The primary objective of this study was to investigate the rate of visual attention and information processing on computerized perceptual speed tasks varying in complexity and familiarity, in similarly educated young and older adults while also accounting for affective factors. All tasks required rapid activation of transient attention, rapid visual processing and conscious perception of the object(s), which can be conceptually related to Posner’s three attentional networks (Posner and Petersen, 1990; Petersen and Posner, 2012). Collectively, the results of the IT and CD tasks indicated that the threshold exposure duration required by older adults to make a correct perceptual judgment was longer than that of younger adults even when the task demands were simple and predominantly requiring alerting and orientation for object recognition, as in the IT task, and even more so when the task was novel, as in the CD task that required new learning, short term memory, a less familiar task strategy and decision making i.e., executive control of attention. By comparison on the semantically more complex FastaReada task that utilized more familiar and regularly practiced skills, older adults demonstrated statistically comparable performance to younger adults, despite requiring complex cognitive skills. Overall, the results from the current study are in line with the *Cattell-Horn* *Theory of Fluid and Crystallized Intelligence* (Cattell, 1963; Horn and Cattell, 1966), Salthouse (1991) *disuse perspective*, the *Processing Speed Theory of Adult Age Differences in Cognition* (Salthouse, 1996) and findings from Madden (2007).

### Age and Performance on Inspection Time Task

Contrary to hypotheses and our previous work (Ebaid et al., 2017), our older adults (60–81 years) required a significantly longer (i.e., slower) estimated threshold exposure time to discriminate and identify simple visual stimuli than did younger adults (18–30 years). Furthermore in this study, a small but significant correlation was exhibited between age and threshold exposure time on the IT task, implying that as age increases, threshold exposure time also increase (i.e., become slower), supporting past research using a similar IT task (Ritchie et al., 2014) and theories of generalized slowing across the healthy lifespan (i.e., Salthouse, 1996). It is important to note that the sample in Ebaid et al. (2017) included a wider age range, with several participants between the ages of 30–50 years in the older group which may be a factor contributing to the discrepancy in findings.

From the viewpoint of the categories of attentional control proposed by Posner and Petersen (1990), the results of the IT task that required rapid activation and orientation of attention to a visual stimulus, with minimal demands on executive control of attention, are in line with those that suggest that older adults demonstrate reduced alerting during ANT tasks, even when controlling for generalized cognitive slowing (Jennings et al., 2007; Kaufman et al., 2016). However, when considering the demands of the IT tasks beyond the main requirement of object recognition, the IT task requires orientation to relevant local stimuli which globally represented a fish, truck or butterfly, and then executive control of attention when deciding which of the three images were presented. Although theoretically the control of attention can be conceptualized as three different networks that carry out functions of alerting, orienting, and executive control, it may not be ideal to dichotomize them when assessing the networks using behavioral measures even if the task is seemingly simple, as in the IT task. Instead, it may be more appropriate to consider the degree to which the attentional networks are required for performance on certain cognitive tasks.

When considering the demands of the IT task and its predominant reliance on bottom-up control of attention, our findings are consistent with those that suggest that older adults are slower and less accurate on task performance reliant on bottom-up processing (Madden, 2007). Although the visual stimuli used in the current study consisted of familiar objects i.e., a fish, truck or butterfly, the figures were made up of many small shapes contributing to the *globally* represented objects (see Figure 1). Thus, it is possible that older participants may have been more distracted by the local figures and hence had more difficulty processing the global image (Oken et al., 1999). Differences in global-local processing between healthy young and older adults have previously been explored in a study conducted by Oken et al. (1999) who reported a significant impairment in the ability of older adults to process global figures compared to local figures. More recent research has also demonstrated that healthy older adults between 65 and 86 years show a significant local-processing bias compared to young and middle-aged adults (Insch et al., 2012). Other studies, however, have reported that older adults are more biased to global processing, and in fact demonstrate global interference compared to young adults when trying to process local figures of small alphabetical letters which globally represent a large incongruent alphabetic letter (Roux and Ceccaldi, 2001).

### Age and Performance on the Change Detection Task

Consistent with predictions, performance on the CD task was significantly different between age groups, with younger adults detecting change between two visual arrays significantly faster than older adults. Furthermore, moderate significant correlations were also exhibited between age and threshold exposure duration on the CD, implicating that as age increases, exposure time required to detect change also increase. Again considering the categories of attentional control (Posner and Petersen, 1990), it may also be the case that deficits in the alerting system demonstrated with aging are associated with more extensive difficulties in orienting and executive control of attention that are required for the CD task: a suggestion put forward by Kaufman et al. (2016). However, as alluded to above, it may be more appropriate to consider the extent or load of task demands on each of the attentional networks, as opposed to explicitly differentiating them when using behavioral measures. More specifically, the task demands for the CD are undoubtedly more complex than those of the IT and have a greater load on executive control of attention, however, both measures still require alerting, orienting, and executive control for accurate task performance, though to different degrees.

Results from the current study are also in line with the underlying presumptions of the Cattell-Horn’s theory (Cattell, 1963; Horn and Cattell, 1966) which suggests that cognitive abilities that involve inductive and deductive reasoning, problem solving, and manipulation of new information, are most susceptible to age-related decline. These findings are also consistent with accuracy-based past studies exploring CD and aging (Costello et al., 2010) and those that report positive relationships between increased age and reaction times in detecting change (i.e., Rizzo et al., 2009). With reference to bottom-up and top-down control of attention, the CD task is heavily reliant on bottom-up processing, and so findings from the current study are also in agreement with those that suggest that bottom-up processing is more susceptible to age-related decline compared to top-down control of attention (Madden, 2007). Furthermore, when there are several components to a visual scene such as hash symbols surrounding each letter in the array (i.e., #A#), and presentation time of stimuli is limited (as in the current study), this often exceeds visual-short term memory capacity (i.e., four-items; Cowan, 2001), which in turn increases task difficulty due to dual-task demands, whereby healthy older adults are often reported to show declines in performance relative to younger adults (Vaportzis et al., 2013). However, when healthy older adults are given adequate time for encoding visual stimuli during dual-task working memory measures such as *N*-back tasks, age-group differences are not observed (see Ebaid and Crewther, 2018). From the perspective of the *Inhibitory Deficit Hypothesis* (Hasher and Zacks, 1988) it is also plausible that the hash symbols surrounding each letter in the array served as distractors to older participants who may not have been as efficient at inhibiting the irrelevant information, therefore impeding overall task performance.

### Age and Performance on FastaReada

Contrary to predictions, there were no significant differences in performance on the FastaReada task between young and older adults, and thus, results are not in line with findings from past research that report an age-related slowing in reading speed (Kliegl et al., 2004; McGowan et al., 2014). Past researchers have also suggested that compared to silent reading, reading aloud is slower given that readers are required to articulate each word and thus, visual fixations remain in the same place for longer (Rayner, 1998, 2009). This suggestion is also persuant to data indicating that talking speed or orofacial movement decline with normal aging (Bilodeau-Mercure et al., 2015). Results from the current study also reject such inferences, however, it is important to note that although in the current study older adults were required to read sentences aloud, we did not measure the speed of verbal articulation, given that the task itself varied in presentation time for each sentence. Specifically, after articulation of a sentence, a button press triggered the exposure of the next six words which became faster or slower depending on each participants’ accuracy performance.

Reading is considered a unique neurobiocultural task (Meyer and Pollard, 2006) which is heavily reliant on rapid processing of visual information and complex cognitive skills including visual attention, continuous inhibition of distractors, continuous access to lexical storage, integration of sublexical, orthographic, phonological, and lexico-semantic information as well as working memory (Cotton and Crewther, 2009; Froehlich et al., 2018) to achieve fast semantic understanding of the text (Daneman and Hannon, 2001; Breznitz and Misra, 2003; Sereno and Rayner, 2003; Blaiklock, 2004; Kinsey et al., 2004; Elhassan et al., 2015). Indeed, fluent reading of text requires alerting, orienting, and executive control of attention for fast and accurate performance, with excess demands on working memory, continuous and rapid access to lexical storage, given that the text was progressively presented for shorter durations. Although the FastaReada is a complex cognitive task reliant on both bottom-up control of visual attention, as well as top-down semantic processing, reading can be considered a familiar and well-practiced task amongst highly educated individuals (Albert and Teresi, 1999; Meyer and Pollard, 2006), which in turn may have had a compensatory effect that enabled older adults to perform as fast as younger participants. When considering that many of the fundamental skills required for fluent reading entail skills such as verbal fluency and vocabulary, results from the current study are consistent with the notion that these skills which comprise *Crystallized Intelligence* remain relatively stable or even improve across the lifespan (Cattell, 1963; Horn and Cattell, 1966; Baltes et al., 1995; Shimamura et al., 1995; Hedden et al., 2005; Elgamal et al., 2011). Furthermore, as participants vision was screened to ensure normal or corrected-to-normal acuity, this to some extent, ensured that sensory deficits in the visual domain were not impeding task performance, especially as stimuli were presented at the same distance each time even though for varying temporal durations.

### Negative Affect and Performance on the IT, CD, and FastaReada in Young and Older Adults

Findings from the current study surprisingly demonstrated that our older adult group reported lower levels of anxiety, depression, and stress symptoms compared to younger adults. Furthermore, there were also no significant correlations between performance on the IT, CD or FastaReada and any category of negative affect for the older adult group. This finding differs from previous research which suggests that older adults typically above 60 years have increased levels of negative affect which in turn is related to performance on cognitive tasks (Beaudreau and O’Hara, 2008, 2009). It is important to note, however, that our sample of older adults were actively engaged in vocational education, and generally active members of their communities, and thus such attributes are likely to contribute to lower levels of negative affect. Supporting this presumption, a study conducted by Glass et al. (2006) found that healthy older adults above 65 who were socially engaged in activities including unpaid community or volunteer work had lower levels of depressive symptoms as measured with the Center for Epidemiologic Studies Depression Scale. In addition, findings from the current study demonstrated that the younger sample had higher levels of depression, anxiety, and stress (though still in the “mild” range) compared to older adults, and these findings are in line with research suggesting that first and second year university students have higher levels of negative affect as measured with the DASS compared to students in more advanced years (Bayram and Bilgel, 2008).

### Limitations

A limitation on the generalizability of findings is the similar education level of the two samples, both of which reported significant tertiary training. Education is a particularly relevant factor, especially for older adults, given that individuals with higher levels of education are often reported to be less susceptible to cognitive decline and forms of dementia later in life (Zhang et al., 1990; Armstrong et al., 2012). Thus, the older adults from the current study who have continued to engage in vocational study post-retirement, may not be representative of the general population of healthy older adults over 60. Future research should aim to include both young and older samples with more diverse levels of education. A further limitation of the current study was the sizeable discrepancy between the numbers of participants in the young adult group compared to the older adults. Indeed, the small sample size in the older adult group may have compromised the statistical power of group-based findings, and so future research should aim to include equal sized samples. Although the overarching aim of our experiment was not to assess differences in cognitive performance between genders, male participants were under-represented in both age-groups. Though there is currently very little robust evidence to suggest gender-differences in cognitive performance in any age-group psychophysically and in neuroimaging (see Hill et al., 2014; Reed et al., 2017; Ritchie et al., 2018), future research may benefit from including an even spread of genders within their study when assessing cognitive performance. In terms of the CD task, more ecologically appropriate stimuli might be better for translational validity using visual scenes of roads or traffic scenarios which may be more familiar to participants, and more applicable to the broader context of biomarkers for safety in driving given that visual deterioration due to presbyopia, glaucoma and age-related maculopathies are common in adults over 60 years of age (Patel and West, 2007; Goertz et al., 2014; Spierer et al., 2016). Clinical assessment of vision and hearing in future studies may enable more rigorous dissociation of sensory receptor decline and cognitive performance if threshold contrast or sound levels are examined, rather than suprathreshold contrasts as used in the current study. Furthermore, in relation to theories which postulate an association between sensory and cognitive decline (Lindenberger and Baltes, 1994; Baltes and Lindenberger, 1997; Schneider and Pichora-Fuller, 2000), normal age-related deficits in vision and hearing may have indirectly affected cognitive performance. Given that both age groups in the current study had very low i.e., less than “mild” levels of depression, anxiety, and stress when measured with the DASS, it may also be useful for future research into visual information processing to include a sample of participants with more variable levels of negative affect in order to more accurately explore its potential impact on cognitive performance.

## Conclusions and Future Directions

The current study demonstrated that threshold rates of visual attention and processing speed are significantly slower for older adults even on perceptual tasks that simply require identification of a familiar visual stimulus, i.e., the IT task. Furthermore, when task demands were increased to measure exposure time required to detect change, older adults were also significantly slower. Interestingly, on the cognitively complex FastaReada which measured the rate of fluent reading, older participants performed comparably to younger adults. Thus, the most obvious temporal difference affecting rate of visual information processing between the three tasks was the bottom-up requirement of the IT and CD tasks for participants to rapidly attend, learn and embed information in short term memory prior to making decisions on visual stimuli identification, or of “change” or “no-change” between two visual arrays. It was also interesting to note that lower levels of depression, anxiety, and stress symptoms were reported for our older adults compared to our younger sample, making it unlikely that negative affect impaired cognitive task performance.

To add further understanding to such findings and the attentional networks proposed by Posner and Petersen (1990), future research should aim to examine underlying biobehavioral mediators of visual attention and rapid perceptual processing, such as eye movements. As eye movements play a significant role in guiding and directing attention (McDowell et al., 2008; Fernandez-Ruiz et al., 2018), it may be valuable to explore these patterns between young and older adults during similar psychophysical tasks particularly given previous suggestions that current tasks used to assess the attentional networks such as the ANT (Fan et al., 2002) may not be reliable in disassociating the networks of attention in aging (MacLeod et al., 2010). The results from the current study are potentially important clinically, in regard to translational clinical tools able to provide rapid non-invasive measures of information processing and visual attention, and should inform theoretical areas of aging research relating to visual attention and processing.

## Ethics Statement

This study was carried out in accordance with the recommendations of the National Statement on Ethical Conduct in Human Research, La Trobe University Human Ethics Committee (UHEC) and all subjects gave written informed consent in accordance with the Declaration of Helsinki. The protocol was approved by La Trobe University Human Ethics Committee, approval number S15/19.

## Author Contributions

SC initiated the project, designed the outline and content, contributed to the writing and data analysis. DE recruited participants, collected and analyzed the data and also contributed to most of the writing, by developing the first and final draft of the manuscript along with SC.

## Conflict of Interest Statement

The authors declare that the research was conducted in the absence of any commercial or financial relationships that could be construed as a potential conflict of interest.

## Footnotes

^1^www.u3a.org.au

^2^www.vpixx.com

^3^http://www.VPixx.com
